# TGFβ and BMP Dependent Cell Fate Changes Due to Loss of Filamin B Produces Disc Degeneration and Progressive Vertebral Fusions

**DOI:** 10.1371/journal.pgen.1005936

**Published:** 2016-03-28

**Authors:** Jennifer Zieba, Kimberly Nicole Forlenza, Jagteshwar Singh Khatra, Anna Sarukhanov, Ivan Duran, Diana Rigueur, Karen M. Lyons, Daniel H. Cohn, Amy E. Merrill, Deborah Krakow

**Affiliations:** 1 Department of Human Genetics, David Geffen School of Medicine at the University of California at Los Angeles, Los Angeles, California, United States of America; 2 Department of Orthopaedic Surgery, David Geffen School of Medicine at the University of California at Los Angeles, Los Angeles, California, United States of America; 3 Department of Molecular, Cell, and Developmental Biology, University of California at Los Angeles, Los Angeles, California, United States of America; 4 Center for Craniofacial Molecular Biology, Ostrow School of Dentistry, University of Southern California, Los Angeles, California, United States of America; 5 Department of Biochemistry and Molecular Biology, Keck School of Medicine, University of Southern California, Los Angeles, California, United States of America; 6 Department of Obstetrics and Gynecology, David Geffen School of Medicine at the University of California at Los Angeles, Los Angeles, California, United States of America; Murdoch Childrens Research Institute, AUSTRALIA

## Abstract

Spondylocarpotarsal synostosis (SCT) is an autosomal recessive disorder characterized by progressive vertebral fusions and caused by loss of function mutations in *Filamin B* (*FLNB)*. FLNB acts as a signaling scaffold by linking the actin cytoskleteon to signal transduction systems, yet the disease mechanisms for SCT remain unclear. Employing a *Flnb* knockout mouse, we found morphologic and molecular evidence that the intervertebral discs (IVDs) of *Flnb*^*–/–*^mice undergo rapid and progressive degeneration during postnatal development as a result of abnormal cell fate changes in the IVD, particularly the annulus fibrosus (AF). In *Flnb*^*–/–*^mice, the AF cells lose their typical fibroblast-like characteristics and acquire the molecular and phenotypic signature of hypertrophic chondrocytes. This change is characterized by hallmarks of endochondral-like ossification including alterations in collagen matrix, expression of Collagen X, increased apoptosis, and inappropriate ossification of the disc tissue. We show that conversion of the AF cells into chondrocytes is coincident with upregulated TGFβ signaling via Smad2/3 and BMP induced p38 signaling as well as sustained activation of canonical and noncanonical target genes *p21* and *Ctgf*. These findings indicate that FLNB is involved in attenuation of TGFβ/BMP signaling and influences AF cell fate. Furthermore, we demonstrate that the IVD disruptions in *Flnb*^*–/–*^mice resemble aging degenerative discs and reveal new insights into the molecular causes of vertebral fusions and disc degeneration.

## Author Summary

Whereas there is a large foundation of knowledge concerning skeletal formation and development, identifying the molecular changes behind Intervertebral Disc (IVD) aging and degeneration has been a challenge. The loss of Filamin B, a protein component of the cell’s cytoskeletal structure, gives rise to Spondylocarpotarsal Synostosis, a rare genetic disorder characterized by fusions of the vertebral bodies. Similarly, mice lacking the Filamin B protein show fusions of the vertebral bodies. We found that these fusions are caused by the early degeneration and eventual ossification of the IVDs. Our study demonstrates that this degeneration is caused by the increase in TGFβ and BMP activity, developmental pathways essential in bone and cartilage formation. These findings represent a significant step forward in our understanding of the molecular basis of IVD degeneration. as well as revealing filamin B’s role in TGFβ/BMP signaling regulation. Moreover, we demonstrate that the study of the rare disease spondylocarpotarsal synostosis in a model organism can uncover mechanisms underlying more common diseases. Finally, our findings provide a model system that will facilitate further discoveries regarding disc degeneration, which affects a significant proportion of the population.

## Introduction

Skeletal dysplasias are a heterogeneous group of more than 450 disorders characterized by abnormalities in patterning, development, and maintenance of the skeleton [1]. Congenital vertebral deformities in some of these disorders, particularly those that lead to progressive fusions, cause chronic pain and deformity and the mechanisms underlying the development of these fusions are poorly understood. Spondylocarpotarsal synostosis (SCT) syndrome is a recessively inherited disorder caused by nonsense mutations in *Filamin B (FLNB);* it is characterized by progressive vertebral, carpal, and tarsal bone fusions, short stature, and scoliosis [2]. The vertebral fusions can occur prenatally, leading to the appearance of an ectopic bar of bone spanning multiple vertebral bodies at birth [3].

Development of the axial skeleton, including the vertebral bodies, occurs via the process of endochondral bone formation, in which a cartilage template is replaced by bone. Endochondral bone growth occurs through the cartilage growth plate, a stratified tissue consisting of reserve, resting, proliferating, and hypertrophic chondrocytes which ultimately undergo apoptosis, leaving voids that become inhabited by osteoblasts forming the primary spongiosa. Vertebral bodies of the axial skeleton develop through endochondral ossification and are separated from each other by intervertebral discs (IVDs). The IVD forms a fibrous joint that functions as a shock absorber for the spine and allows for slight flexion. Each IVD consists of two functional domains: the inner gel-like center of the nucleus pulposus and the outer fibrous tissue of the annulus fibrosus (AF). At the interface between the IVD and the vertebral body lies the endplate, a transitional tissue of mineralized matrix surrounding hypertrophic-like chondrocytes.

The process of endochondral ossification requires the coordination of multiple signaling pathways. TGFβ/BMP signaling is essential for early cartilage and bone formation as well as postnatal growth [4, 5]. The BMP and TGFβ pathways signal via their canonical receptor phosphorylated Smads (R-Smads) [6, 7]. The TGFβ pathway signals through R-Smads 2 and 3 while the BMP pathway utilizes R-Smads 1, 5, and 8. Upon ligand binding the receptors phosphorylate and activate the R-Smads. The activated R-Smads translocate to the nucleus and form complexes with additional proteins to induce or repress target gene transcription. Both pathways also utilize the MAPK/ERK and TAK1/p38 pathways as non-canonical signaling pathways. Misregulation of TGFβ and BMP signaling affects many aspects of skeletal development, including formation of the IVD [8], and over-activation of these pathways has been shown to cause ectopic bone formation in several tissues [9–11]. The IVD AF, as well as cartilage, ligament, and tendon tissues, are derived from progenitor pools consisting of distinct cell populations with unique expression profiles. While TGFβ and BMP signaling are critically important in musculoskeletal tissues, studies have recently shown that each of these distinct cell populations responds differently to changes in these respective signaling pathways [12].

Filamins are cytoskeletal proteins that stabilize actin filament networks and link them to the cellular membrane, thus forming a scaffold for integrating cell mechanics and signaling [13]. Filamins have also been shown to interact directly with Smads 2 and 3, as well as 1 and 5, central mediators of canonical TGFβ and BMP signaling, respectively [14, 15]. FLNB is expressed throughout the cartilage growth plate as well as in the cartilaginous condensations of developing vertebrae [16]. Biallelic loss of function mutations leading to loss of FLNB cause SCT, while heterozygosity for missense mutations in *FLNB* produces a spectrum of autosomal dominant skeletal disorders including boomerang dysplasia (OMIM 112310); Larsen syndrome (OMIM 150250); and atelosteogenesis I and III (AOI, OMIM 108720; AOIII, OMIM 108721).

In this study we used mice homozygous for a gene trapped *Flnb* allele (*Flnb*^*–/–*^) [17]. We previously showed that *Flnb*^*–/–*^mice phenocopy SCT and show progressive vertebral, carpal, tarsal, and sternal fusions as well as smaller overall body size, making this an ideal animal model for studying progressive vertebral fusions [17]. Mice heterozygous for the gene trap are unaffected. FLNB is expressed in the developing skeleton, throughout the growth plate, in the developing limb buds and between the neural arches of the vertebrae [17] as well as in the somites, which include the cells destined to become the vertebral bodies and IVDs of the spine. It was initially hypothesized that SCT vertebral fusions resulted from failure to segment the vertebrae properly [18, 19], but analysis of the *Flnb* knockout mouse model demonstrated that the vertebrae form normally but subsequently fuse [17]. In this study, we seek to explain how absence of FLNB produces spinal fusions, the most compelling phenotypic finding in both humans and mice. We demonstrate that in the absence of FLNB, TGFβ and BMP signaling is increased both *in vitro* and *in vivo*, demonstrating that FLNB is required for the attenuation of TGFβ/BMP signaling. We also show that the mutant AF undergoes ectopic differentiation toward a chondrogenic lineage in the IVD, leading to abnormal endochondral ossification. The phenotypic findings resemble those seen in aging disc degeneration and therefore provide a developmental model for degenerative disc disease.

## Results

### Absence of FLNB causes progressive fusions and abnormalities in the postnatal vertebral growth plate and IVD

**Fig 1:**
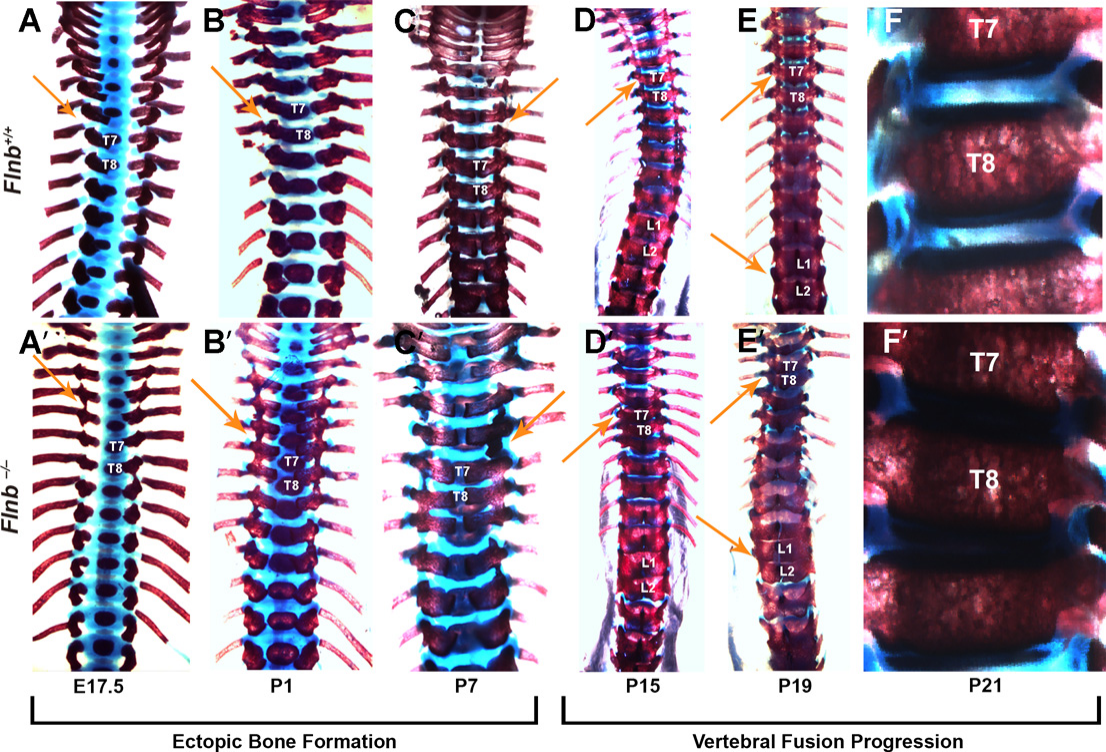
*Flnb*^*–/–*^vertebrae progressively fuse in the thoracic and lumbar areas of the spine. Posterior view of mouse spines stained for cartilage proteoglycans (blue) and mineralized bone (red). (A-C, A′-C′) The *Flnb*^*–/–*^mouse spine exhibited ectopic ossifications between the neural arches of the thoracic area (arrows). (D, D′) P15 image depicts representative vertebral fusions between thoracic vertebrae in the *Flnb*^*–/–*^mouse spine (arrows). (E, E′) At P19, vertebral fusions and ectopic ossifications have progressed into the lumbar area (arrows). (F, F′) Anterior view. Multiple discs have disappeared and mineralized to bone in the P21 *Flnb*^*–/–*^mouse spine. N = 3 for each timepoint.

In SCT patients, vertebral abnormalities are seen early in infancy, frequently presenting as scoliosis before eventually progressing to the fusion phenotype. Our previous study demonstrated initially normal formation of the IVD, but did not determine the precise time points at which ectopic ossifications and fusions occur. To investigate the spinal phenotype, *Flnb*^*–/–*^and *Flnb*^*+/+*^ skeletal preparations were stained with alcian blue (to stain cartilage proteoglycans) and alizarin red (to stain mineralized bone) (Fig 1). Ectopic bone formations are seen between the neural arches of the thoracic region of the spine beginning at E17.5 and progressing through P7 (Fig 1A–1C, 1A′–1C′, arrows). These formations likely represent the ossification of the interspinous ligaments. Development of thoracic vertebral fusions (T7, T8, and T9) began later, at P5, and progressed as shown in P15 vertebrae (n = 5) (Fig 1D and 1D′). Lumbar vertebrae (T13, L1, and L2) are nearly completely fused by P19 (Fig 1E and 1E′). By P21 IVDs have completely disappeared at the thoracic and lumbar fusion sites (Fig 1F and 1F′). These observations show that the spinal phenotype is indeed progressive and begins in the thoracic area of the spine before progressing to the lumbar vertebrae.

**Fig 2:**
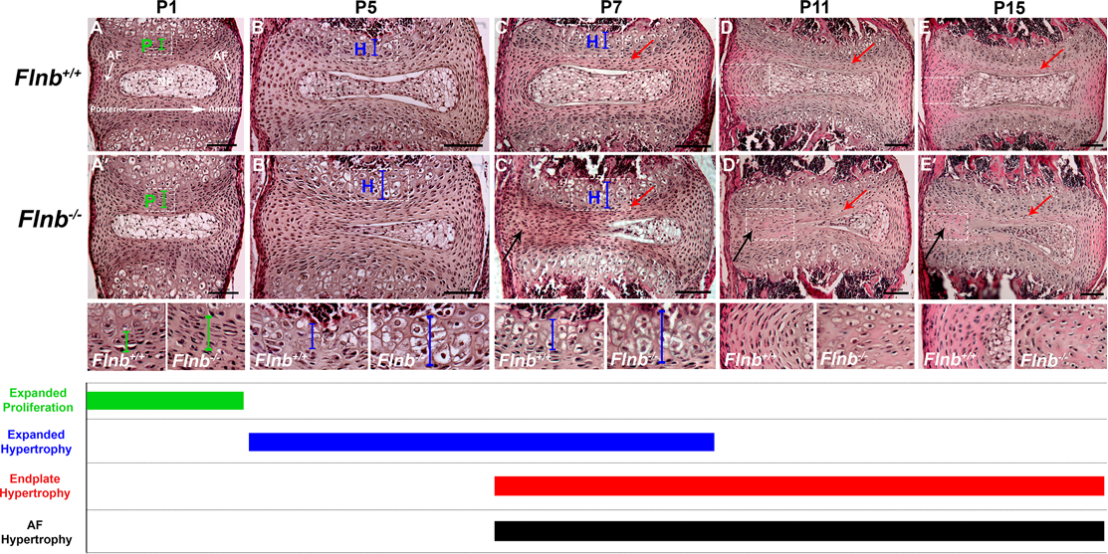
Absence of FLNB progressively affects vertebral growth plate and IVD tissue morphology. H&E staining of (A-E) *Flnb*^*+/+*^ and (A′-E′) *Flnb*^*–/–*^T7 thoracic IVDs and vertebral growth plates. Left: posterior, Right: anterior. (A, A′) *Flnb*^*–/–*^P1 vertebral growth plates showed an enlarged proliferative zone compared with *Flnb*^*+/+*^ (green brackets). (B, B′) *Flnb*^*–/–*^P5 vertebral growth plates showed enlarged hypertrophic zones compared with *Flnb*^*+/+*^ (blue brackets). (C, C′) *Flnb*^*–/–*^P7 vertebral growth plates showed increased hypertrophic zones (blue brackets). *Flnb*^*–/–*^P7 AF tissues exhibited rounder cell morphologies (black arrows) at the same time point as the initiation of endplate mineralization (red arrows). (D, D′, E, E′) *Flnb*^*–/–*^P11 and P15 AF tissues exhibited similar morphology (black arrows) to the now mineralized endplate tissues (red arrows).

We demonstrated FLNB expression in the AF and nucleus pulposus of E14.5 mice via X-gal staining, indicating that FLNB plays a role in the development of these tissues (S1 Fig). Histological analysis of sagittal IVD sections from P1 to P15 showed that the IVDs developed apparently normally until P1 but IVD disruptions were evident by P5 (Fig 2). Within the *Flnb*^*–/–*^vertebral body growth plates there was an initial increase in size of the proliferative zone at P1, followed by an increased zone of hypertrophic chondrocytes at P5 and P7 (Fig 2A′–2C′) as compared with control animals (Fig 2A–2C). By P15 this increase in hypertrophy was less evident as the vertebral growth plate reached its final stage of differentiation (Fig 2E and 2E′). In addition, the vertebral body heights were significantly shorter in *Flnb*^*–/–*^mice (S2 Fig). This was likely due to the accelerated chondrocyte differentiation observed in the growth plate that removed chondrocytes from the proliferative pool, resulting in decreased longitudinal growth. Investigation of the nucleus pulposus showed that beginning at P5, the nucleus pulposus became compressed and the disruption became more severe as the IVD aged as demonstrated in P7, P11, and P15 IVDs (Fig 2B′–2E′).

### Absence of FLNB induces ectopic differentiation towards chondrogenic lineages in AF tissue

**Fig 3:**
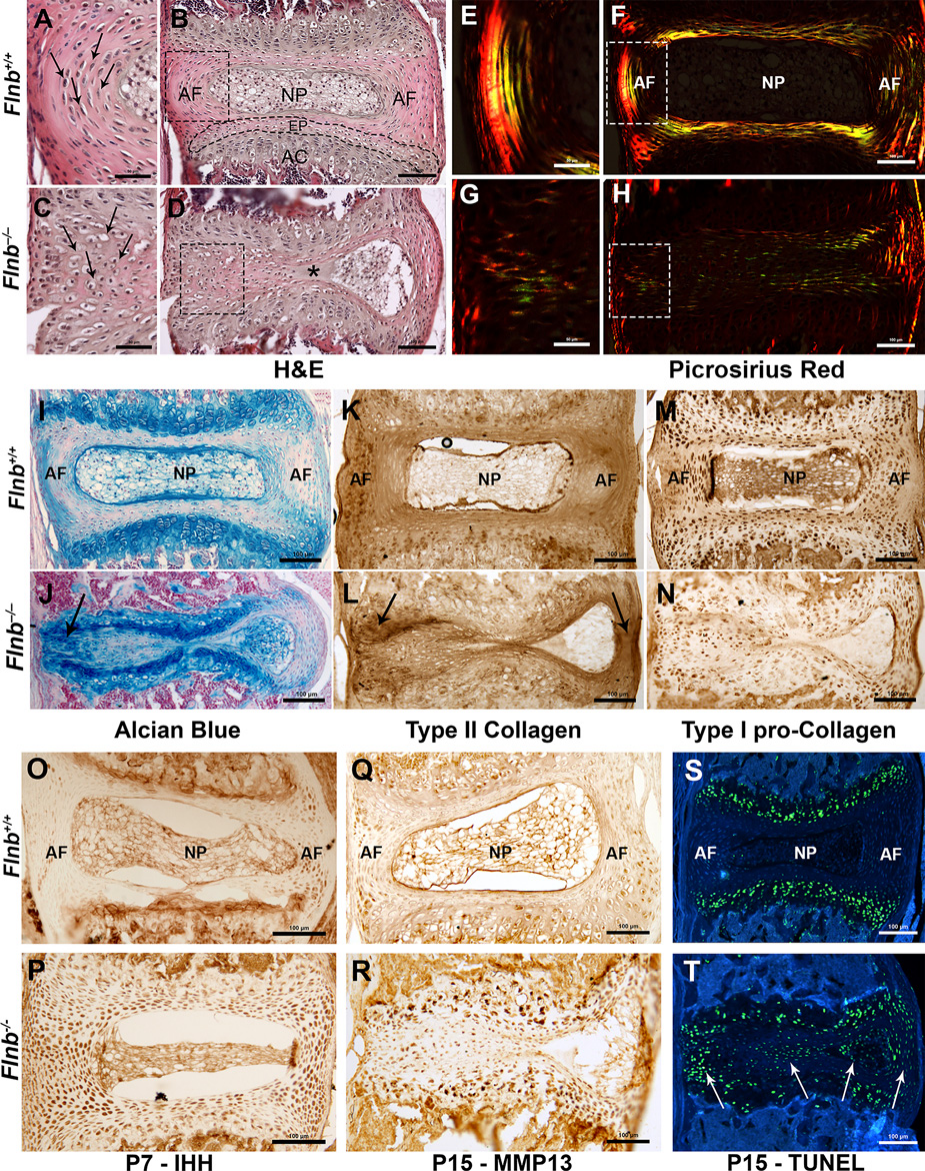
*Flnb*^*–/–*^IVDs exhibit disruptions in AF cell morphology; show altered ECM and markers of enhanced chondrocyte differentiation. Left: posterior, Right: anterior. (A-D) H&E staining of P15 T7 IVDs. The IVD is composed of: NP = Nucleus Pulposus, AF = Annulus Fibrosus, EP = Endplate, AC = Articular Cartilage. (A-C) P15 *Flnb*^*+/+*^ AF cells had a fibroblast-like appearance while *Flnb*^*–/–*^AF cells were enlarged and exhibited more hypertrophic-like qualities (arrows). (D) The *Flnb*^*–/–*^IVD showed a disruption of the AC hypertrophic zone as well as a fissure within the NP/AF boundary (shown by *). (E-H) Polarized imaging of picrosirius red staining of P15 mouse IVD paraffin sections. (E) *Flnb*^*+/+*^ IVDs exhibited a distinct structure of collagen bundles forming the lamellae of the AF. (G) The *Flnb*^*–/–*^collagenous matrix of the posterior AF lacked an organized structure. (I,J) P15 disc tissue stained with alcian blue. The posterior AF of the *Flnb*^*–/–*^disc contained increased proteoglycan deposition compared with *Flnb*^*+/+*^ (black arrow). (K,L) IHC against type II collagen. Type II collagen was increased in the anterior and posterior AF of the *Flnb*^*–/–*^IVD (black arrows). (M,N) IHC against type I pro-collagen. Pro-type I collagen protein expression was decreased throughout the AF, end-plate and nucleus pulposus of the *Flnb*^*–/–*^IVD. (O,P) IHC against IHH in P7 T7 IVD. The *Flnb*^*–/–*^disc contained increased IHH protein expression in the AF compared with *Flnb*^*+/+*^. (Q,R) IHC against MMP13 in P15 T7 IVD. The *Flnb*^*+/+*^ AF exhibited some MMP13 expression in both the posterior and anterior AF regions. MMP13 protein expression was increased throughout the AF and endplate in the absence of FLNB. (S,T) TUNEL staining of P15 T7 IVD to detect apoptotic activity. *Flnb*^*+/+*^ discs exhibit apoptosis almost exclusively in the hypertrophic zone of the vertebral body growth plate. In *Flnb*^*–/–*^discs, the apoptotic zone seen in *Flnb*^*+/+*^ discs was no longer present and there was an increase in apoptotic cells visible within the posterior and anterior AF as well as the nucleus pulposus. Blue background represents auto-fluorescence and is shown for orientation only.

Using sagittal sections of the vertebral spine in both *Flnb*^*+/+*^ and *Flnb*^*–/–*^, we observed starting at P7 the cells of the AF begin to exhibit a change in morphology by becoming rounder and more hypertrophic in appearance as demonstrated in P7, P11, and P15 IVDs (Fig 2C–2E, 2C′–2E′, black arrows). These findings suggest that a process of cell transformation occurred in the AF cells of *Flnb*^*–/–*^mouse spines rather than solely the encroachment of the existing growth plate. A closer examination of P15 IVDs in Fig 3A–3D revealed that *Flnb*^*–/–*^mice exhibited a developing front of chondrocyte-like cells and an expanding acellular matrix that overtook the cells of the nucleus pulposus leading to a fissure (Fig 3D, asterisk). This expanding front of cells led to a narrowing of the nucleus pulposus (Fig 3D). In the posterior AF of *Flnb*^*–/–*^IVDs, the cells became rounder, resembling the hypertrophic-like cells normally found in the cartilaginous endplate (Fig 3C, arrows).

Because hypertrophy is associated with changes in matrix composition, we examined AF matrix structure and composition [20]. Loss of polarized birefringence visualized with picrosirius red staining indicated a severe disruption of the collagen structure with the fibers becoming thinner and more irregular in the *Flnb*^*–/–*^IVD, indicating matrix disorganization (Fig 3E–3H). Additionally, the *Flnb*^*–/–*^mouse IVD spaces exhibited markedly increased alcian blue staining indicating greater proteoglycan content (Fig 3I and 3J, arrow). To further investigate the *Flnb*^*–/–*^IVD matrix, we probed for specific collagen distributions and demonstrated an increase in type II collagen staining in the outer AF (Fig 3K and 3L, arrows) as well as a decrease in type I collagen staining in the AF of *Flnb*^*–/–*^mice compared with *Flnb*^*+/+*^ (Fig 3M and 3N). This change in the type II: type I collagen ratio, as well as an increased proteoglycan content, is indicative of a change from a fibrous to a more cartilaginous matrix.

**Fig 4:**
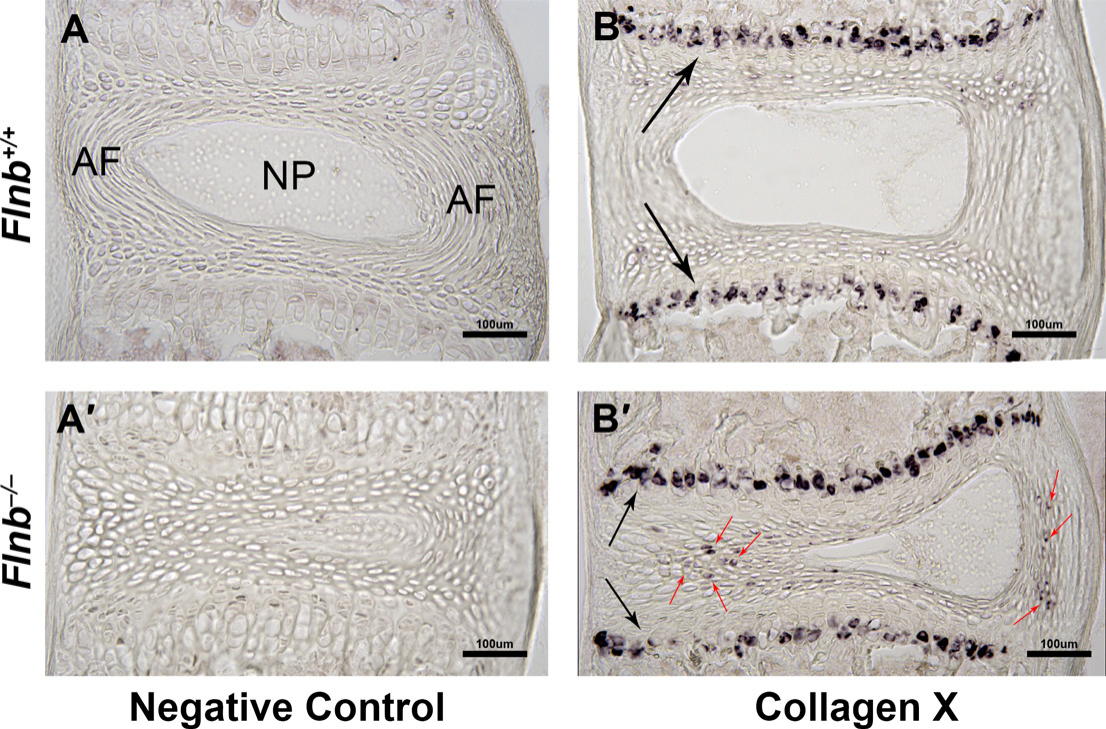
*Flnb*^*–/–*^AF tissues exhibit Collagen X expression. Left: posterior, Right: anterior. (A, A′, B, B′) RNA *in situ* staining of P15 T7 IVD sagittal sections. (A, A′) *Flnb*^*+/+*^ and *Flnb*^*–/–*^*in situ* sections using sense riboprobe as a negative control. (B) *Flnb*^*+/+*^ *in situ* section using antisense riboprobe against ColX. ColX expression is exhibited in the hypertrophic zone of the *Flnb*^*+/+*^ vertebral body growth plate (black arrows) and not in the IVD. (B′) *Flnb*^*–/–*^*in situ* section using antisense riboprobe against ColX. ColX expression is exhibited in both the vertebral body growth plate hypertrophic zone (black arrows) as well as the transformed hypertrophic *Flnb*^*–/–*^AF cells (red arrows).

To investigate whether there were cell fate changes in the AF cells, we examined the AF and nucleus pulposus regions of *Flnb*^*–/–*^mouse spines for expression of the chondrocyte specific markers Indian Hedgehog (IHH) for prehypertophic chondrocytes and Matrix Metalloproteinase-13 (MMP13) for hypertrophic chondrocytes. There was an early increase in IHH expression within the AF of P7 *Flnb*^*–/–*^IVDs (Fig 3O and 3P). We also observed a subsequent increase in MMP13 expression throughout *Flnb*^*–/–*^P15 IVDs (Fig 3Q and 3R) compared with *Flnb*^*+/+*^. These findings are consistent with the gradual inappropriate and premature differentiation of AF cells to a hypertrophic chondrocyte phenotype. Additionally, we carried out TUNEL staining to detect apoptotic activity characteristic of terminally differentiated hypertrophic chondrocytes (Fig 3S and 3T). Whereas in the *Flnb*^*+/+*^ section, apoptotic activity is restricted to the hypertrophic region of the vertebral growth plate (Fig 3S), there is evidence of increased apoptotic activity in the AF as well as the nucleus pulposus of *Flnb*^*–/–*^IVDs (Fig 3T, arrows). We also performed RNA *in situ* hybridization on frozen sagittal sections of the mouse spine at P15 using a Collagen X (*ColX*) riboprobe to detect *in vivo ColX* expression, a known specific marker of hypertrophic chondrocytes. While the *Flnb*^*+/+*^ IVD showed *ColX* expression restricted to the hypertrophic region of the vertebral body growth plate, the *Flnb*^*–/–*^IVD showed *ColX* expression within the AF of the collapsing IVD (Fig 4). These data confirms the transformation of fibroblastic AF cells to hypertrophic chondrocytes in *Flnb*^*–/–*^IVDs.

**Fig 5:**
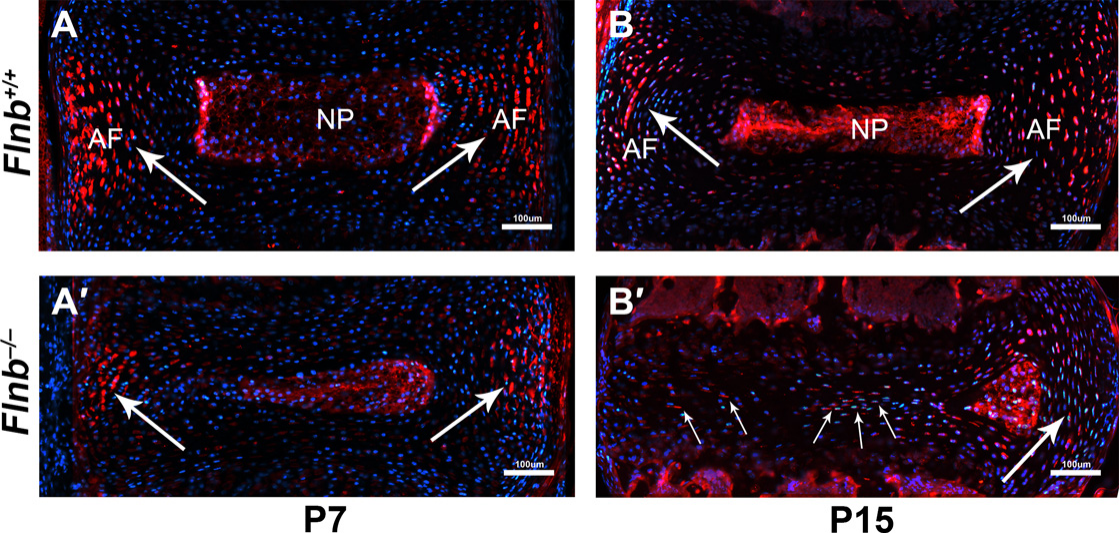
Transformed hypertrophic AF cells continue to express AF marker Scleraxis in *Flnb*^*–/–*^IVDs. (A-D) IHC analysis of Scleraxis-GFP expression in P15 mouse disc tissue paraffin sections. (A,B) *Flnb*^*+/+*^ IVDs at P7 and P15 show SCX expression in the AF tissues (white arrows). (A′,B′) *Flnb*^*–/–*^IVDs at P7 and P15 exhibit continued SCX expression in the transformed hypertrophic AF cells.

Although we have shown that AF cells adopt gene expression patterns of hypertrophic chondrocytes in the *Flnb*^*–/–*^IVDs, we further sought to determine if these findings result from transformation of AF cells as opposed to invasion by chondrocytes. Thus, we crossed *Flnb*^*–/–*^mice with mice containing a GFP reporter driven by the *Scleraxis (Scx)* promoter. SCX expression is used as a marker for tendons, ligaments, and AF tissue, but not chondrocytes [21]. Using paraffin embedded spine sections, we stained for GFP expression in the IVD. At ages P7 and P15, the *Flnb*^*+/+*^ IVDs showed SCX expression in the AF tissues (Fig 5A and 5B, white arrows). Importantly, SCX expression was also observed in the inappropriately transformed hypertrophic cells of *Flnb*^*–/–*^IVDs at P7 and P15 (Fig 5A′ and 5B′, white arrows). Co-expression of SCX in cells that also express genetic markers of hypertrophic chondrocytes indicated that AF cells become hypertrophic chondrocytes. These findings show that the AF cells differentiate from a fibroblast-like cell to prehypertrophic then hypertrophic-like chondrocytes, leading to destruction of the nucleus pulposus through apoptosis.

### FLNB attenuates TGFβ signaling pathway activation in the annulus fibrosus

**Fig 6:**
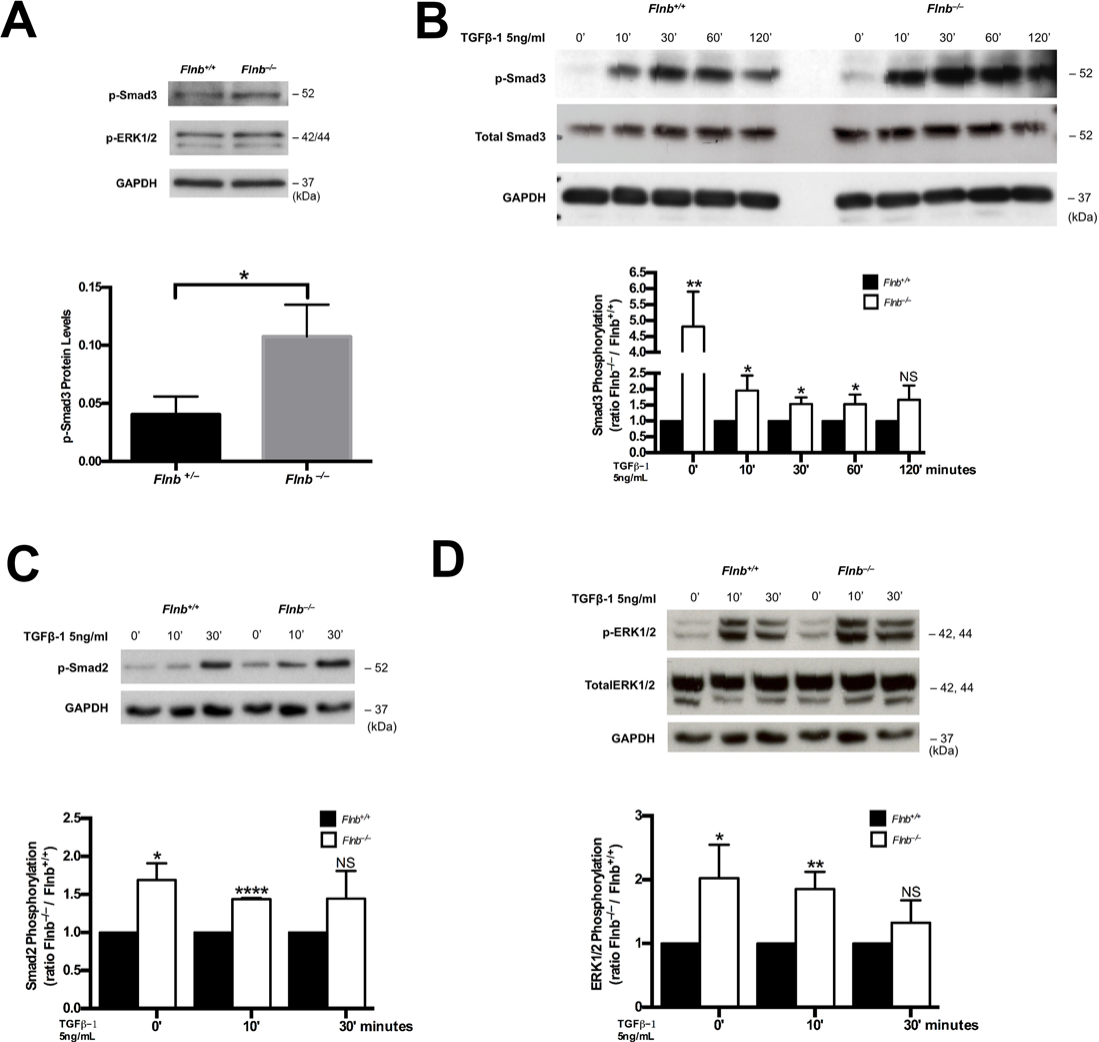
TGFβ signaling increased in absence of FLNB *in vitro* and *in vivo*. (A) Western blot analysis of protein lysates extracted from AF tissue of P15 pups. (B-D) Western blot analysis of protein lysates extracted from cultured primary mouse chondrocytes. (B) P-Smad3 and p-ERK levels are significantly increased both with and without TGFβ-1 (5ng/mL) stimulation in mutant cells whereas total Smad3 levels are unchanged, N = 6. (C) P-Smad2 levels are significantly increased both with and without TGFβ-1 stimulation in mutant cells, N = 6. (D) P-ERK levels are significantly increased endogenously as well as after 10 minutes of TGFβ-1 stimulation whereas total ERK levels remain unchanged, N = 6.

**Fig 7:**
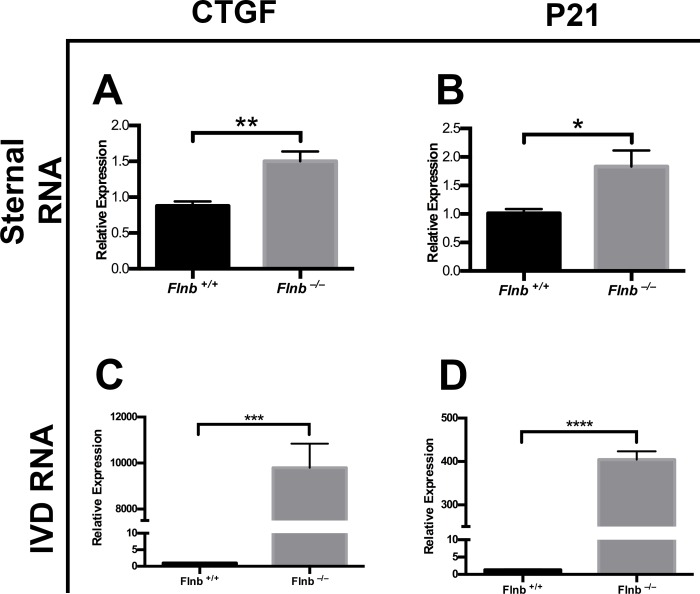
TGFβ nuclear target RNA expression increased in absence of FLNB *in vivo*. (A,B) RT-qPCR analysis results using RNA derived from P1 mouse sternums. The expression levels of the TGFβ nuclear targets *CTGF* and *p21*, are both significantly increased in *Flnb*^*–/–*^mouse sternums, N = 6. * = p<0.05, ** = p<0.01, data are represented as mean ± SEM. (C,D) RT-qPCR analysis results using RNA derived from P15 mouse IVDs. The expression levels of the TGFβ nuclear targets, *CTGF* and *p21*, are significantly increased in *Flnb*^*–/–*^mouse IVDs, N = 3. *** = p<0.001, **** = p<0.0001, NS = not significant, data are represented as mean ± SEM.

Since FLNB has been shown to interact with Smad3, a component of canonical TGFβ signaling in cultured primary chondrocytes [15], we chose to investigate whether there are further changes in the TGFβ signaling pathway in chondrocytes and the IVD. Protein extracts from *Flnb*^*–/–*^IVDs demonstrated increased endogenous levels of phospho-Smad3 and phospho-ERK in the AF compared with *Flnb*^*+/+*^ (Fig 6A). The sterna of *Flnb*^*–/–*^mice, similar to vertebrae, also exhibit fusions [17] and sternal chondrocytes were used to further interrogate the TGFβ pathway. To determine the *in vitro* responsiveness of *Flnb*^*–/–*^cells to TGFβ pathway activation, primary sternal chondrocytes were isolated from P1 *Flnb*^*+/+*^ and *Flnb*^*–/–*^mice and stimulated with TGFβ-1 ligand. In both unstimulated and stimulated *Flnb*^*–/–*^primary chondrocytes, TGFβ signaling activity was upregulated as measured by increased phosphorylation of Smad3 (Fig 6B), as well as Smad2 (Fig 6C). Additionally, with TGFβ-1 stimulation, we found increased phospho-ERK signaling indicating a noncanonical pathway signaling response (Fig 6D) [22]. To further define the increase in TGFβ signaling activity, we quantified the *in vivo* expression of TGFβ signaling targets including *Connective Tissue Growth Factor (CTGF)* and *p21*, both of which are highly expressed in cartilage, by qPCR of RNA derived from *Flnb*^*+/+*^ and *Flnb*^*–/–*^P1 sternal cartilage as well as P15 IVD AF tissue [23–25]. *Flnb*^*–/–*^sternal cartilage showed a statistically significant increase in *Ctgf* (Fig 7A) and *p21* expression (Fig 7B). We also observed statistically significant increases in both *Ctgf* (Fig 7C) and *p21* (Fig 7D) in P15 IVDs indicating that the observed increase in TGFβ signaling activation also results in increases in TGFβ target transcription *in vivo*.

### Loss of FLNB up-regulates BMP mediated TAK1/p38 signaling in the postnatal IVD affecting homeostasis

**Fig 8:**
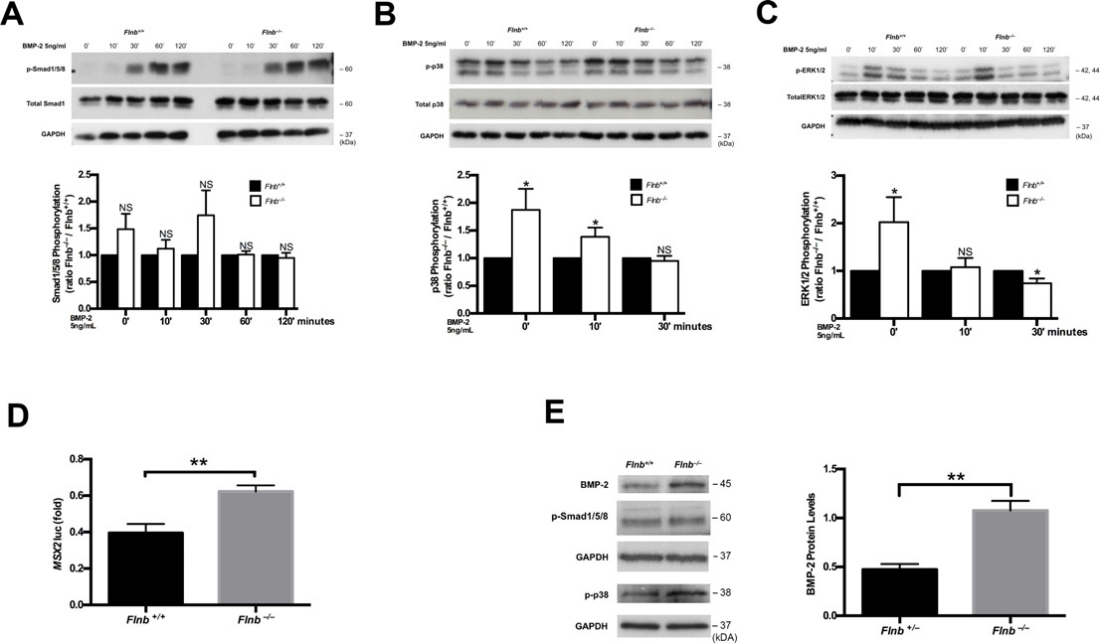
BMP non-canonical pathway activation is increased in the absence of FLNB *in vivo* and *in vitro*. (A-C) Western blot analysis of protein lysates extracted from cultured primary mouse chondrocytes. (A) P-Smad1,5,8 levels remain unchanged in *Flnb*^*–/–*^when compared with *Flnb*^*+/+*^, N = 6. (B) P-p38 levels are increased endogenously as well as upon BMP-2 (10ng/mL) stimulation in mutant versus *Flnb*^*+/+*^ chondrocytes, N = 3. (C) p-ERK levels are upregulated endogenously but are subsequently decreased in mutant chondrocytes following 30 minutes of BMP-2 stimulation. N = 4. (D) Luciferase assay measuring *Msx2* promoter activity in primary mouse chondrocytes. *Msx2* promoter activity is increased in *Flnb*^*–/–*^chondrocytes, N = 3. * = p<0.05, ** = p<0.01, data are represented as mean ± SEM. (E) Western blot analysis of protein lysates extracted from AF tissue of P15 pups. BMP-2 and p-p38 protein expression levels are increased in *Flnb*^*–/–*^IVDs with no change in p-Smad1/5/8 levels, N = 7.

The BMP signaling pathway is an inducer of chondrocyte differentiation. Therefore, observed ectopic ossification of IVDs seen in *Flnb*^*–/–*^mice suggested a possible increase in BMP signaling activation. Similar to the *in vitro* TGFβ experiments, we stimulated primary sternal chondrocytes with BMP-2 ligand. Although the data indicated a trend towards increased phosphorylation of receptor Smad1,5,8, we did not see a statistically significant change in treated mutant versus *Flnb*^*+/+*^ primary sternal chondrocytes (Fig 8A). We therefore tested whether there was an alteration in non-canonical signaling within the mutant chondrocytes. We detected a significant increase in the phosphorylation of p38 in both unstimulated and BMP-2 stimulated mutant sternal chondrocytes (Fig 8B). A previous study by Liu et al. demonstrated that increases in BMP activated TAK1/p38 result in inhibition of the MAPK/ERK pathway [26]. We therefore tested whether the increase in p-p38 in our mutant chondrocytes exhibited the same inhibitory effect. Similar to the TGFβ stimulation experiments, *Flnb*^*–/–*^cells have increased p-ERK at baseline (Fig 8C), adding complexity to the *in vitro* analyses of p-ERK. However, although there was an endogenous increase of ERK phosphorylation levels in *Flnb*^*–/–*^chondrocytes, these levels were no longer increased after 10 minutes and significantly decreased after 30 minutes of BMP-2 stimulation in mutant versus *Flnb*^*+/+*^ chondrocytes (Fig 8C). Our finding supports that increased BMP-2 activation of p38 results in inhibition of ERK phosphorylation and shows non-canonical up-regulation of the BMP pathway in the absence of FLNB. The Liu et al. study also demonstrated that p38 inhibition of ERK had the ability to increase Smad1 nuclear activity and localization [26]. To determine if there was augmented pathway activation through increased p-p38 and decreased ERK, we performed a *Msx2* luciferase assay in primary sternal chondrocytes using a p-Smad1,5 reporter construct to test for potential increased R-Smad1,5,8 nuclear activity. *Msx2* is a direct target of Smad mediated BMP signaling [27]. We observed an endogenous increase of *Msx2* transcription in *Flnb*^*–/–*^chondrocytes compared with *Flnb*^*+/+*^ (Fig 8D). Further, to determine if BMP signaling pathway targets are inappropriately activated in *Flnb*^*–/–*^IVDs, we interrogated by western blot analysis whole tissue IVD lysates for expression of BMP-2, a target of the pathway, as well as p-Smad1/5/8 and p-p38 levels. There was a statistically significant increase in BMP-2 expression and an increase of p-p38 in the IVDs of *Flnb*^*–/–*^mice with no change in p-Smad1/5/8 levels (Fig 8E). Increased total BMP-2 and *Msx2* transcription are consistent with increased BMP pathway output.

**Fig 9:**
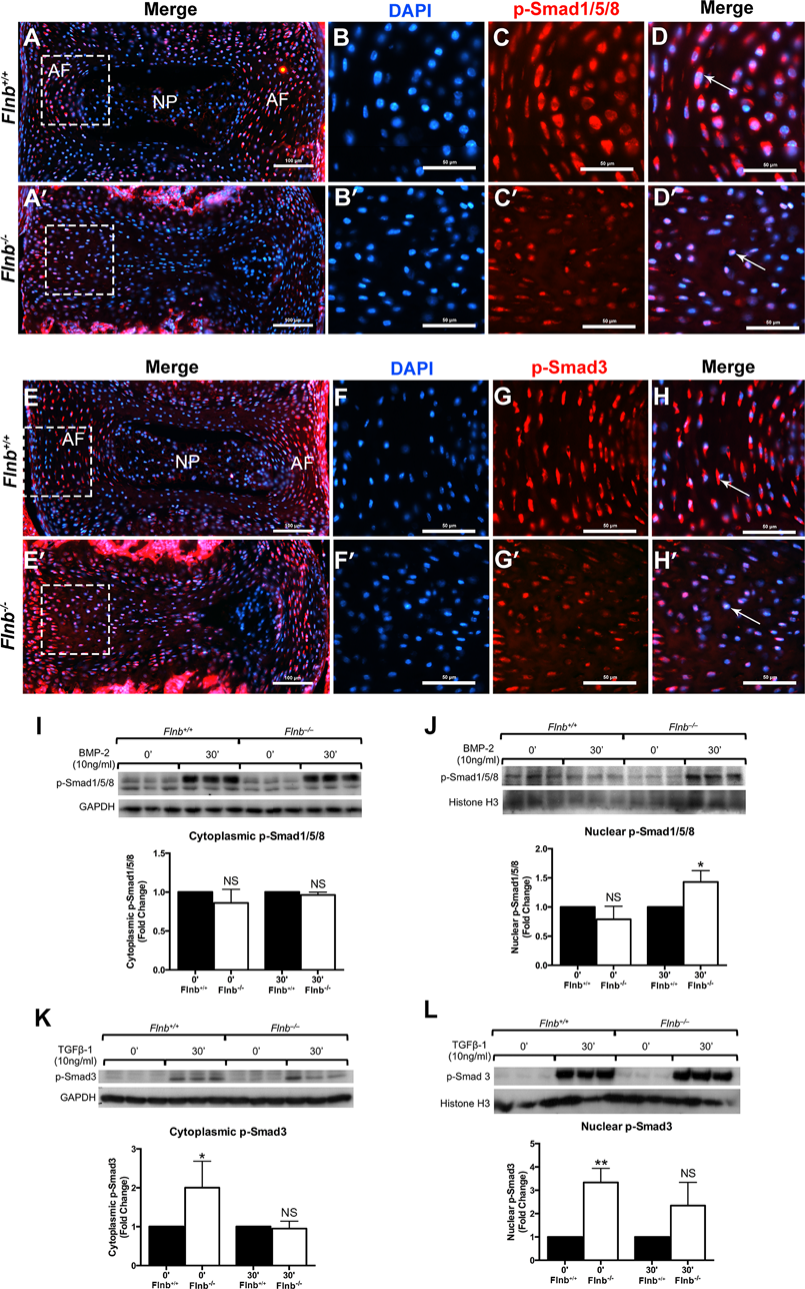
TGFβ and BMP pathway components exhibit nuclear localization in *Flnb*^*–/–*^AF. IHC analysis of P15 mouse disc tissue paraffin sections. (A, A′, E, E′) Whole IVDs shown for orientation. (B, B′, F, F′) DAPI channel only showing nuclear staining. (C, C′, G, G′) Red channel only showing protein localization. (D) P-Smad1,5,8 (red) has a cytoplasmic localization in the *Flnb*^*+/+*^ AF. (D′) P-Smad1,5,8 (red) exhibits a nuclear localization as it co-localizes with the nucleus (blue) In the AF of *Flnb*^*–/–*^mice. (H) P-Smad3 (red) has a cytoplasmic localization in the *Flnb*^*+/+*^ AF. (H′) P-Smad3 (red) exhibits a nuclear localization as it co-localizes with the nucleus (blue) in the AF of *Flnb*^*–/–*^mice. (I-L) Western blot analysis of fractionated protein lysates extracted from cultured primary mouse sternal chondrocytes. (I,J) p-Smad1,5,8 levels remain unchanged in the cytoplasm but are significantly higher in the nuclei of stimulated primary chondrocytes. (K,L) p-Smad3 levels are significantly upregulated in both the cytoplasm and the nucleus without stimulation. (K,L) N = 3. NS = Not Significant, * = p<0.05, ** = p<0.01, data are represented as mean ± SEM.

We subsequently tested whether an increase in Smad1 nuclear localization can be directly visualized in the AF of *Flnb*^*+/+*^ and *Flnb*^*–/–*^mice. In the *Flnb*^*+/+*^ discs, p-Smad1,5,8 proteins were found in both the cytoplasm and the nucleus of AF cells (Fig 9A–9D, arrow). However, in the *Flnb*^*–/–*^AF, p-Smad1,5,8 predominantly demonstrated nuclear localization (Fig 9A′–9D′, arrow) with very little cytoplasmic staining. Consistent with our earlier TGFβ experiments, we demonstrated the same increased nuclear localization of p-Smad3 in *Flnb*^*–/–*^AF cells (Fig 9E–9H, 9E′–9H′, arrows). To confirm these findings we determined nuclear versus cytoplasmic levels of p-Smad1,5,8 and p-Smad3 by western blot analysis of stimulated primary chondrocytes (Fig 9I–9L). As previously demonstrated, this data set also showed an increase in both cytoplasmic and nuclear p-Smad3 (Fig 9K and 9L). Similar to the previous BMP-2 stimulated *in vitro* experiments, total cytoplasmic p-Smad1,5,8 levels were unchanged in *Flnb*^*+/+*^ versus mutant chondrocytes, yet the nuclear fraction showed increased Smad1,5,8 phosphorylation (Fig 9I and 9J). Taken together, these findings are consistent with a model in which the levels of canonical BMP signaling are not impacted by loss of *Flnb*, but the increase in non-canonical phosphorylation of p38 results in increased Smad1 nuclear activity due to the inhibition of ERK.

## Discussion

Our *Flnb* knockout model shows that absence of FLNB leads to progressive fusions of the thoracic and lumbar vertebrae with patterns directly phenocopying those seen in SCT patients. We focused on the spine because it is the most severely affected region of the skeleton in both SCT patients and *Flnb*^*–/–*^mice. Our results indicate that the spinal fusions in *Flnb*^*–/–*^mice initiate in the thoracic vertebrae before progressing into the lumbar region. Within the *Flnb*^*–/–*^cartilage growth plates of the vertebral body flanking the IVD space, there was an initial expansion of proliferative chondrocytes at P1, followed by an expansion of the hypertrophic zone at later stages. This change in growth plate morphology suggests that the growth plate chondrocytes undergo an accelerated differentiation cycle in the absence of FLNB, as has been seen in the growth plates of appendicular elements [28].

**Fig 10:**
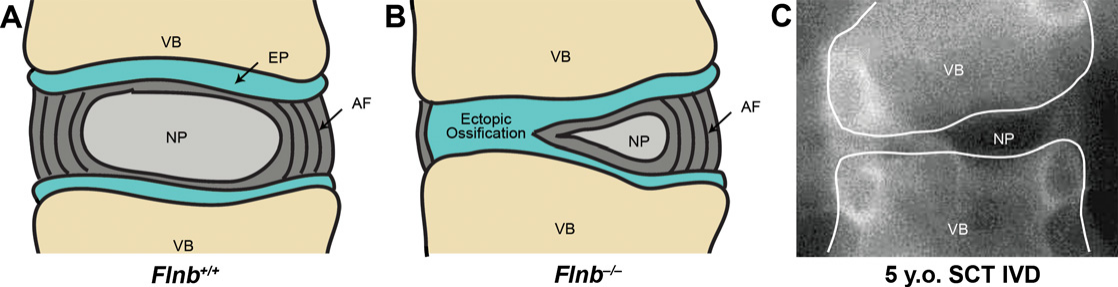
Illustration of tissue morphology change in *Flnb*^*–/–*^IVD. (A) *Flnb*^*+/+*^ IVD illustration. (B) *Flnb*^*–/–*^IVD illustrating transition of AF to hypertrophic-like state resembling the mineralized endplate. Flanking vertebral bodies shift in position as IVD degenerates resulting in inappropriate spinal curvature. (C) IVD of 5 year old SCT patient. Positions of flanking vertebrae suggest compression of the IVD identical to those observed in *Flnb*^*–/–*^mouse spines.

Further, in the posterior AF of *Flnb*^*–/–*^IVDs, the cells are rounder and enlarged, resembling the cartilaginous cells normally found only in the endplate. The findings of altered collagen composition and structure, along with increases in sulfated proteoglycans, type II collagen, IHH, MMP13 expression, *ColX* expression, and apoptosis are indicative of advancing chondrocyte maturation in *Flnb*^*–/–*^AF. The transforming *Flnb*^*–/–*^cells also continue to express the AF marker SCX, showing that the observed hypertrophic cells are inappropriately transformed AF cells. This suggests that the AF cells in the IVD of *Flnb*^*–/–*^mice undergo an inappropriate transition from fibroblast-like to chondrocytic cells, culminating in transition to terminally differentiated chondrocytes that undergo apoptosis. Whereas MMP13 is used as a marker for late hypertrophic chondrocytes, it also plays a role in matrix degradation as it degrades collagens and cleaves aggrecan [29]. While its’ inappropriate expression in the AF may be due to the changes in AF cell fate, the consequences of its expression likely results in matrix disruption and decreased ability of the IVD to absorb mechanical forces. It is likely that changing cellular characteristics and the disruption of the extracellular matrix (ECM) reduces the ability of the IVD to absorb mechanical stress, resulting in the collapse of the IVD. The final consequence of these changes is that the IVD transitions through endochondral ossification to bone, producing vertebral fusions (Fig 10). In our model, the IVD fusions progress posteriorly to anteriorly and these changes in IVD morphology are temporal with spinal fusions and disappearance of the IVD by P21.

TGFβ/BMP signaling has long been implicated in ectopic bone formation and the combination of both TGFβ and BMP up-regulation produces more ectopic bone formation than BMP up-regulation alone [30]. Our findings of increased canonical and noncanonical TGFβ signaling supports previous findings by Zheng et al. that demonstrated up-regulated TGFβ signaling through increased p-Smad3 in a different *Flnb*^*–/–*^mouse model that also exhibited vertebral fusions [15]. Supporting our findings of enhanced TGFβ signaling activity, we showed increased *in vivo* RNA expression of the TGFβ transcriptional targets *Ctgf* and *p21*. p21 protein acts as an inhibitor of cyclin-dependent kinase CDKs 2, 3, 4, and 6 and participates in cell cycle inhibition through the TGFβ pathway. Its up-regulation is associated with increased hypertrophic differentiation in chondrocytes [31, 32]. The premature onset of chondrocyte hypertrophy observed in the vertebral growth plates of *Flnb*^*–/–*^mice may result from premature cell cycle exit coupled with accelerated differentiation caused by increased p21 expression. Additionally, *Hu et al*. described a decrease in CDK phosphorylation as well as enhanced chondrocyte differentiation after FLNB knockdown, which supports our data [28]. Interestingly, a study conducted by Sohn et al. elegantly demonstrated (using a knockdown model of the TGFβ receptor TGFβR2 under the control of a Col2a1cre promoter) that the consequences of loss of TGFβ signaling in the embryonic sclerotome results in disruption of IVD development [8]. This study showed that TGFβ signaling is essential to IVD embryonic development and our findings further show the importance of TGFβ signaling on postnatal IVD homeostasis.

BMP signaling has been consistently implicated as one of the primary inducers of chondrocyte differentiation. We did not observe increased p-Smad1,5,8 levels in response to BMP-2 stimulation either *in vitro* or *in vivo*. However, BMP-2 stimulation did increase BMP activated p38 phosphorylation, a component of TAK1/p38 non-canonical signaling. Further, in *Flnb*^*–/–*^chondrocytes after BMP-2 stimulation there was diminished ERK phosphorylation at 30 minutes consistent with previous work that showed p38 functions in the inhibition of the MAPK/ERK pathway (Liu at al., 2012). This p38 induced inhibition of ERK results in increased Smad1 nuclear activity and localization [26]. Our findings of enhanced *in vitro* activation of *Msx2*, a direct target of BMP Smad mediated signaling [27] and increased nuclear localization of p-Smad1,5,8 supports that in the absence of FLNB, there is activation of the BMP pathway through the p38 and subsequent inhibition of ERK. The aforementioned *Hu et al*. study showed no change in p-ERK levels in their ATDC5 cell model. This difference may result in part from different outcomes in knockout versus knock-down models.

FLNB is expressed throughout the developing skeleton [17], yet the increase in TGFβ/BMP signaling has a profound effect specifically on the AF and the vertebrae growth plate. The vertebral body growth plate, endplate and annulus fibrosus are all derived from the same mesenchymal tissues arising in the sclerotome [33]. The morphological fate of these tissues is governed by the differential levels and ratios of pathway activation and gene expression. A recent study showed that cartilage, ligament/tendon, and IVD tissues develop from distinct progenitor pools that express Sox9, an SRY related transcription factor that is important in the regulation of cartilage development, and/or Scleraxis (Scx), a basic helix-loop-helix transcription factor highly expressed in tendons and ligaments as well as the IVD [12, 21]. Whereas tendon progenitors are mainly derived from Scx^+^/Sox9^–^ progenitor pools, short ligaments and the IVD AF are derived from Scx^+^/Sox9^+^ progenitor pools. Cartilage is derived from Scx^–^/Sox9^+^ progenitor cells. An additional study showed that knockdown of BMP signaling in Scx^+^/Sox9^+^ (ligament/IVD) expressing progenitor cells inhibits tissue development and differentiation indicating that Scx^+^/Sox9^+^ expressing cells are more sensitive to changes in TGFβ/BMP signaling whereas Scx^–^/Sox9^+^ and Scx^+^/Sox9^–^ expressing progenitor cells are less sensitive [34]. In human SCT as well as our mouse model, the affected tissues include cartilage, ligaments, and the IVD. We have demonstrated FLNB expression in the developing annulus fibrosus and nucleus pulposus of E14.5 mice, implying that the absence of FLNB may have a more profound effect on these Scx^+^/Sox9^+^, TGFβ/BMP sensitive cell populations. In our model, the persistent up-regulation of TGFβ/BMP signaling occurring throughout postnatal development of the axial skeleton is likely to disrupt the normal molecular balance in signaling and induce a change in cell fate. These findings suggest that a crucial role of FLNB is to regulate and attenuate TGFβ/BMP signaling in these tissues in order to maintain normal morphological boundaries during postnatal tissue development.

In the postnatal IVD, it is vital that signaling and matrix integrity be maintained within the disc, and degeneration is caused by a loss of homeostasis. In aging disc degeneration, the cells of the AF go from a spindle shape to a more rounded chondrocyte-like shape [35], collagen fibers become thinner and more irregular [36], and IVDs exhibit a fissure between the nucleus pulposus and AF boundary as well as increased apoptotic activity within the nucleus pulposus and the AF [37]. An increase in MMP13 production has also been shown in degenerated discs and identified as one of the causative factors in the reorganization of the matrix due to its tendency to cleave aggrecan and collagens [29]. These morphologic and molecular findings are those seen in the IVDs of *Flnb*^*–/–*^mice. Previous work in aging disc degeneration has shown increases in BMP and TGFβ signaling [35, 38]. Additionally, patients harboring activating Smad3 mutations exhibited increased Smad3 phosphorylation levels coupled with disc degeneration beginning as early as 12 years of age [39]. These findings further support that inappropriate and increased TGFβ and BMP signaling due to the absence of FLNB is phenotypically and molecularly similar to aging degenerating discs and suggests that our model may be useful in dissecting the molecular mechanisms underlying this common disease process.

### Conclusions

In our study, we demonstrated that loss of FLNB in a mouse models produces an alteration in AF cells from a fibrous to a cartilaginous fate and disruption of the nucleus pulposus, leading to degeneration, subsequent collapse of the disc followed by fusion of the flanking vertebral bodies. This phenotype results from inappropriate up-regulation of the TGFβ canonical and BMP non-canonical pathways. Previous studies have interpreted that the observed increases in TGFβ/BMP signaling within degenerative discs occur as an effort to repair the extracellular matrix [40]. Because elevated p-Smad2 and 3 were observed in chondrocytes prior to IVD disc degeneration, our model suggests that increased TGFβ and BMP signaling as a consequence of loss of FLNB initiates the cascade of cell fate changes including differentiation, subsequent IVD degeneration and bony fusions. This study has revealed the cascade of some molecular mechanisms governing the development of vertebral fusions and postnatal disc maintenance and degeneration, providing insights which may direct studies that lead to rationale targets for therapeutic solutions.

## Materials and Methods

### Generation of mice

Mice used in these experiments were generated and characterized previously [17]. Briefly, The 129/Ola ES cell line (BayGenomics RRF239) contained the gene-trap vector pGT0Lxf inserted into intron 3 of *Flnb*. The gene trap contains a splice acceptor site and a β-gal Neomycin cassette causing loss of functional protein expression and production of a short fusion transcript. The cells were microinjected into C57BL/6 blastocysts and chimeras were mated with C57BL/6 mice to generate *Flnb*^*+/–*^mice. The mice used in this study were maintained on a mixed genetic background. *Scx/GFP-Cre* mice were a generous gift from the Ronen Schweitzer laboratory.

### Histological analyses and immunohistochemistry

Tissues were fixed in 10% neutral buffered formalin, decalcified using Immunocal decalcification solution and then paraffin embedded. Paraffin blocks were sectioned sagitally at 5–10 μm, and stained with Alcian Blue/Nuclear Fast Red, Hematoxylin/Eosin, and Picrosirius Red. Additional paraffin sections were stained for apoptotic activity using the In Situ Cell Death Detection Kit, Fluorescein (Roche). Sections used for staining were taken from the middle of the spine and each staining and IHC protocol was repeated with at least three biological replicates and three technical replicates for each biological replicate. Whole skeletal preparations were prepared and stained with Alcian Blue and Alizarin Red as previously described [41].

Staining protocols: For Alcian Blue/Nuclear Fast Red staining, deparaffinized and rehydrated sections were incubated in 3% acetic acid solution and subsequently stained in 10% Alcian Blue (Sigma, A5268)/3% Acetic Acid solution. This was followed by counter staining in a 0.1% Nuclear Fast Red (Sigma, N8002)/5% Aluminum Sulfate (Sigma, A7523) solution. For Hematoxylin/Eosin staining, deparaffinized and rehydrated sections were stained with Hematoxylin QS (Vector H-3404), rinsed in tap water and then destained in 0.5% Acid EtOH. Sections were then counterstained with a 0.1% Eosin Y (Sigma E4009)/90% EtOH/0.5% Glacial Acetic Acid solution. For Picrosirius Red staining, deparaffinized and rehydrated sections were stained in a 0.1% Direct Red 80 (Sigma, 43665)/Saturated Picric Acid (Sigma, P6744) solution followed by counterstaining Hematoxylin QS. Additional paraffin sections were stained for apoptotic activity using the In Situ Cell Death Detection Kit, Flourescein (Roche, 11684795910).

For immunohistochemistry, paraffin sections were boiled for 20 minutes in Antigen Unmasking Solution (Vector) and subsequently stained using a Rabbit Specific HRP/DAB (ABC) Detection IHC Kit (Abcam). Beta-galactosidase staining was carried out on 10 mm thick frozen sections following fixation in 10% PBF for two minutes and three washes in 1x PBS. Sections were stained for beta-gal activity using X-gal in the staining solution (5 mM K3Fe(CN)6,5 mM K4Fe(CN)6, 2 mM MgCl2, 1 mg/mL X-gal made in 1x PBS) at 37°C overnight. After washing twice in PBS the slides were counterstained with nuclear fast red (N3020, Sigma) and mounted. All experiments were performed with at least three biological replicates and four sections per replicate.

Primary Antibodies used for IHC: Phospho-Smad3 (Cell Signaling, cs 9520, 1:50), Phospho-Smad1/5/8 (Cell Signaling, cs 9511, 1:25), Collagen II (Abcam, 34712, 1:50), Pro-Collagen I (Santa Cruz, 8787, 1:50), MMP13 (Abcam, 39012, 1:25), Indian Hedgehog (Abcam, 39634, 1:50), and GFP (Abcam, ab290, 1:100).

### RNA *in situ* hybridization

P15 mouse spines were dissected, immediately placed in 4% paraformaldehyde, and rotated at 4°C for three days. Tissue was washed in PBS at 4°C for thirty minutes, then transferred to 0.48M EDTA DEPC treated pH 7.4 decalcification solution and rotated at 4°C for three days. Samples were checked via X-ray for proper decalcification and, if necessary, decalcification solution was replaced and samples were rotated for an additional day. Samples were transferred to 30% sucrose/PBS solution and rotated at 4°C overnight. Samples were finally embedded in OCT compound.

Riboprobes were generated using the DIG RNA labeling kit (Roche, 111750259100). Probes were purified using an RNEasy mini kit (Qiagen, 74104). 16 μm sagittal spine sections were collected on slides and fixed for 10 minutes in 4% formaldehyde in PBS. All steps were performed at room temperature unless otherwise noted. Sections were washed 3×3 minutes in PBS, immersed in 0.2M HCl for 10 minutes, washed 2×5 minutes in PBS, immersed in Proteinase K buffer for 10 minutes (40ug/mL proteinase K, 6.25mM EDTA in 0.05M Tris, pH 7.5), washed 2×5 minutes in PBS, immersed in 4% paraformaldehyde for 20 minutes, washed 2×5 minutes in PBS, immersed in Acetic Anhydride buffer for 10 minutes (0.1M triethanolamine pH8.0 + 1/400 acetic anhydride), washed 5 minutes in PBS, washed with 2x SSC for 5 minutes, dehydrated, then incubated with hybridization buffer for at least 5 mins (50% formamide, 5x SSC, 5x Denhardt’s solution, 0.25 mg/mL baker’s yeast RNA, 0.5 mg/mL single stranded fish sperm DNA). DIG labeled probes were diluted to 1–4 ug/ml in hybridization buffer and applied to sections, which were then covered with plastic coverslips and incubated in a humidified chamber containing 50% formamide/5x SSC for 18–20 hours at 52°C. Sections were washed in 35% Formamide/5X SSC/0.1% Tween-20 for 15 minutes at 52°C 3 times, then washed in 50% Formamide/2X SSC at 52°C for 15 minutes 3 times, then washed in Maleic Acid Buffer (0.1M Maleic Acid, 0.15M NaCl, pH 7.5) + 1% Tween-20 (MABT) 10 minutes at room temperature 2 times, then blocked in 1X MABT + 10% goat serum + 2% BMB (blocking agent, Roche) for 1 to 4 hours. Sections were incubated with 1:2500 anti-DIG AB-Alkaline phosphatase ALP conjugated antibody for DIG (Roche) in 1X MABT + 2% goat serum + 1% BMB overnight at 4°C in a humidified chamber.

Sections were washed with MABT 15 minutes 4-5X + levamisole (0.048g/100mL), then washed with Detection buffer (0.1M Tris-HCl, 0.1M NaCl pH 9.5) + 0.1% TWEEN for 10 minutes 3 times, then slides were immersed in Color Reaction reagent (300ul NBT/BCIP (Roche, cat# 11175041910) in 15mL Detection buffer) and left in the dark at room temperature until color appears. Sections were washed in PBS for 5 minutes, washed in water for 5 minutes, and mounted with Hydro-Matrix.

### Cell culture and tissue extraction

Mouse primary chondrocytes were extracted from the ribcages of P1 mice. Ribcages were digested in 1mg/ml Type II Collagenase (Life Technologies)/serum free DMEM solution (GIBCO) for two hours in a 37°C/5% CO_2_ incubator. The liberated cells were subsequently filtered through a 40um sterile cell strainer and plated in DMEM + 10% FBS (GIBCO). Primary chondrocytes were stimulated at multiple time points using recombinant human TGFβ-1 (R&D, 10ng/ml) ligand and then lysed in RIPA buffer. Six biological replicates were performed for stimulation experiments. Cytoplasmic and nuclear fractions were isolated using the Subcellular Protein Fractionation Kit for Cultured Cells (Thermo Scientific). Three biological replicates were performed for stimulated cell fractionation experiments.

IVD discs were dissected from P15 mouse spines in PBS using a dissecting microscope. IVDs were placed in DMEM + 10% FBS (GIBCO) until dissections were complete. Discs were then washed with PBS, transferred to a 15mL conical tube containing a 10mL solution of DMEM + 0.03% Type II Collagenase (Life Technologies) and incubated overnight in a 37°C/5% CO_2_ incubator. The next day, the Collagenase was neutralized with DMEM +10% FBS, the cells collected by centrifugation at 1,000 rpm for 7 minutes, washed with PBS and the solution was passed through a 40um filter to removed larger particulates. For protein lysates, after centrifugation again at 1,000 rpm for 7 minutes, the resulting cell pellet was resuspended in RIPA buffer supplemented with phosphatase inhibitors (Sigma, P0044) and protease inhibitors (Sigma, P8340). We used discs T5 through T11 for protein analysis.

### Western blot analysis

Stimulated primary chondrocytes were rinsed with phosphate buffered saline. The monolayer cells in each well were lysed in RIPA buffer supplemented with phosphatase inhibitors (Sigma, P0044) and protease inhibitors (Sigma, P8340). Both AF tissue and primary chondrocyte lysates were incubated at 4°C for 30 minutes and centrifuged for 10 minutes at 10,000 rpm. The protein concentration was determined using a BCA protein assay, and equivalent amounts of protein (20 μg) were separated by electrophoresis on 10% SDS-polyacrylamide gels and transferred onto polyvinylidene fluoride membranes. After blocking for 1 hour with 5% milk in Tris-buffered saline-Tween (TBST), membranes were incubated with primary antibodies in 3% BSA/TBST solution at 4°C with gentle shaking overnight. Membranes were incubated with horseradish peroxidase-conjugated secondary antibody at a concentration of 1:2000 at room temperature for 1 hour and detected using an ECL plus kit (Cell signaling, 7071). The band intensities were demonstrated to be in the linear range and their intensities were captured using a digital image scanner, quantified using imageJ (NIH, Bethesda, MD) and the data subjected to statistical analysis.

Primary Antibodies used for Western Blots: Phospho-Smad3 (Cell Signaling, cs 9520, 1:1000), Smad3 (Cell Signaling 9523, 1:1000), Phospho-Smad1/5/8 (Cell Signaling, cs 9511, 1:1000), Smad1 (Cell Signaling, 9743, 1:1000), Phospho-Erk p44/42 MAPK (Cell Signaling, cs 9101, 1:1000), Erk p44/42 MAPK (Cell Signaling 9102, 1:1000), Phospho-p38 (Cell signaling, cs 9211, 1:1000), Beta-Actin (Cell Signaling, cs 4967, 1:1000), BMP2 (Abcam, ab 141933, 1:750), GAPDH (Cell Signaling, cs 2118, 1:1000), and Histone-H3 (Abcam, 1:1000).

Each primary chondrocyte cell experiment was repeated with at least 6 biological replicates. *Flnb*^*+/+*^ and *Flnb*^*–/–*^IVD protein lysates were derived from three pooled IVD tissue samples each containing six IVDs from each of three mice. Quantified bands were normalized to housekeeping gene levels (GAPDH). Because western blots for each biological replicate were performed separately, *Flnb*^*–/–*^samples were analyzed as ratios against *Flnb*^*+/+*^ samples, *Flnb*^*+/+*^ samples were at a value of 1. Data were analyzed by Student’s T-test; the results are shown as the mean ± standard error of a given number of trials (n) as noted in the Fig legend. P ≤ 0.05 was considered statistically significant.

### RT-qPCR

RNA was extracted from isolated mouse sternal tissue and IVD AF tissue using TRIzol reagent (Life Technologies). cDNA was prepared from 1 ug of RNA using RevertAid First strand cDNA synthesis kit (Thermo Scientific) and amplified using Maxima SYBR Green/ROX qPCR Master Mix. Expression levels were calculated using the 2^deltaCT-method of analysis against the stable housekeeping gene beta-2-microglubulin (B2M) [42]. Significance was determined via Student’s T-test. Biological replicates were performed six times each for sternal tissue and three times each for AF tissue with three technical replicates.

Primer Sequences: B2M (Forward) tggtgcttgtctcactgacc; (Reverse) tatgttcggcttcccattct, CTGF (Forward) CTGCTATGGGCCAGGACT; (Reverse) CGTCACACACCCACTCCTC; MMP13 (Forward) GGA CAA GCA GTT CCA AAG GC; (Reverse) CTTCATCGCCTGGACCATAAAG; p21 (Forward) CGGTGGAACTTTGACTTCGT; (Reverse) CACACAGAGTGAGGGCTAAGG.

### Ethics statement

UCLA institutional animal care and use committee (IACUC) Approval Number 2010-082-13C. Animals underwent euthanasia with isoflurane and then cervical dislocations as approved by the institutional review board.

## Supporting Information

S1 Fig FLNB is expressed in the developing IVD. Distribution of FLNB in the E14.5 mouse IVD. (A) FLNB expression (blue stain) in a sagittal spinal section of E14.5 embryos. FLNB is expressed in the early developing annulus fibrosus (white arrows), nucleus pulposus (red arrow), and strongly in the developing vertebral body tissues (VBC). (B) Negative control for the X-gal stain of an E14.5 mouse sagittal section. Nuclei are stained red, VBC = Vertebral Body Cartilage.
(TIF)

S2 Fig *Flnb*^*–/–*^vertebral bodies exhibit decreased height. Cleared P15 vertebral bodies stained stained for cartilage proteoglycans (blue) and mineralized bone (red). *Flnb*^*–/–*^vertebral bodies exhibit decreased height when compared with *Flnb*^*+/+*^. N = 3, ** = p<0.01.
(TIF)

S3 Fig Negative and IgG controls for immunohistochemistry. Left: posterior, Right: anterior. (A, A′) Negative control IHC in P15 T7 IVD. (B, B′) IHC using rabbit whole IgG. (C, C′) IHC using goat whole IgG.
(TIF)
