# Supplementary figures and images for: TGFβ and BMP Dependent Cell Fate Changes Due to Loss of Filamin B Produces Disc Degeneration and Progressive Vertebral Fusions
Source: PLoS Genet. 2016 Mar 28;12(3):e1005936. doi: 10.1371/journal.pgen.1005936 (PMC4809497; doi:10.1371/journal.pgen.1005936)

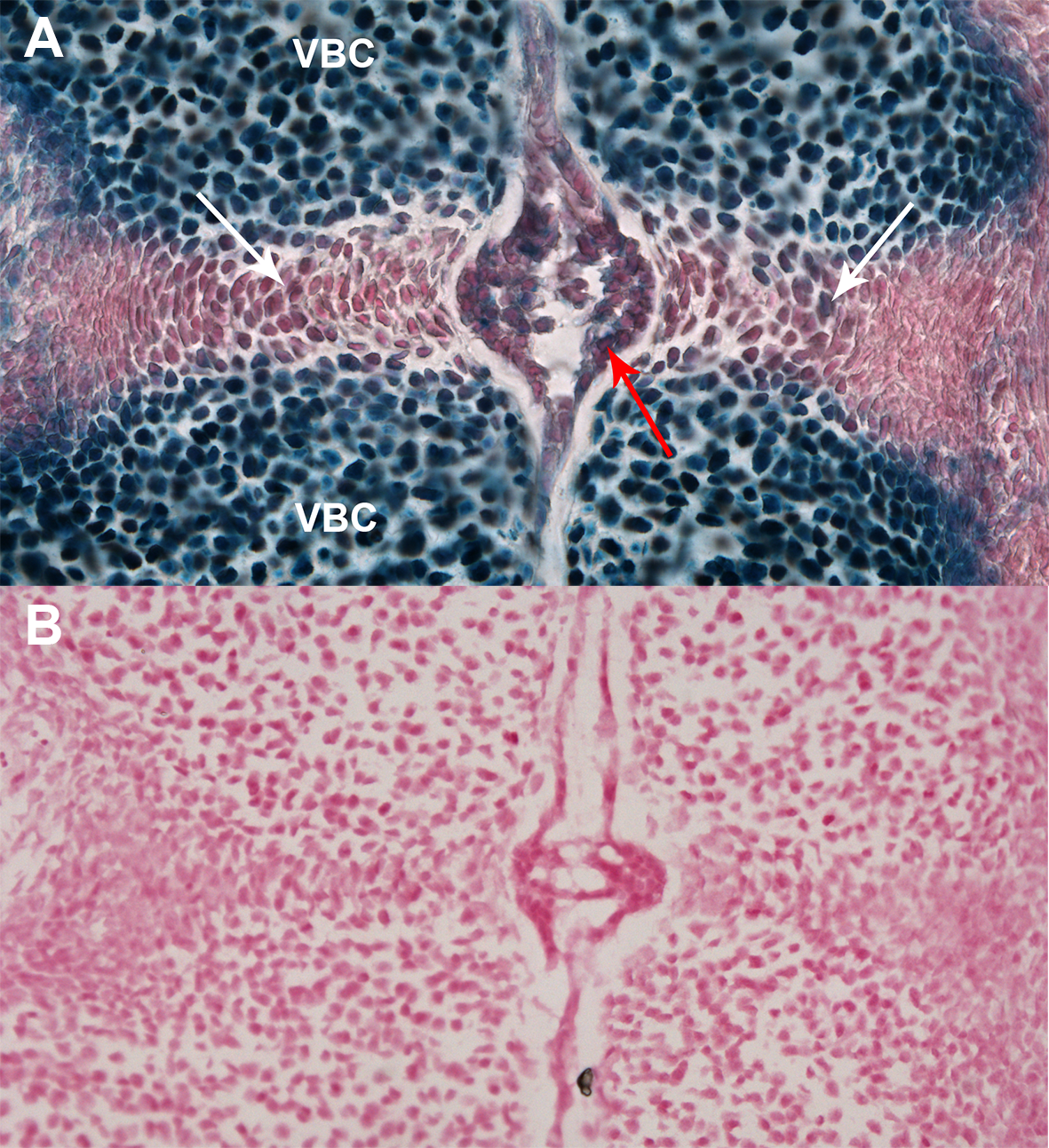

Supplement: S1 Fig — Distribution of FLNB in the E14.5 mouse IVD. (A) FLNB expression (blue stain) in a sagittal spinal section of E14.5 embryos. FLNB is expressed in the early developing annulus fibrosus (white arrows), nucleus pulposus (red arrow), and strongly in the developing vertebral body tissues (VBC). (B) Negative control for the X-gal stain of an E14.5 mouse sagittal section. Nuclei are stained red, VBC = Vertebral Body Cartilage. (TIF) [file pgen.1005936.s001.tif]

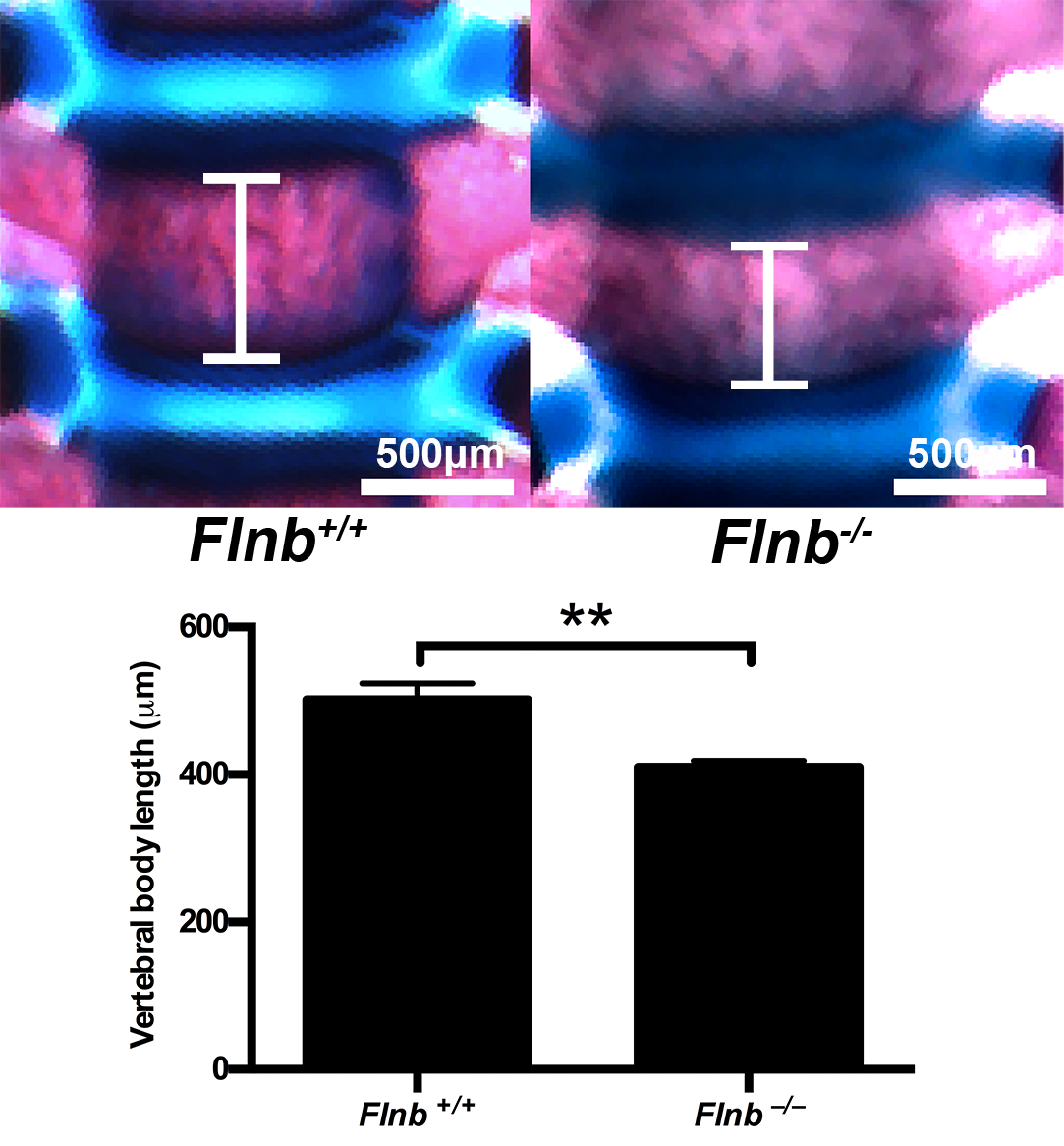

Supplement: S2 Fig — Cleared P15 vertebral bodies stained stained for cartilage proteoglycans (blue) and mineralized bone (red). Flnb–/–vertebral bodies exhibit decreased height when compared with Flnb+/+. N = 3, ** = p<0.01. (TIF) [file pgen.1005936.s002.tif]

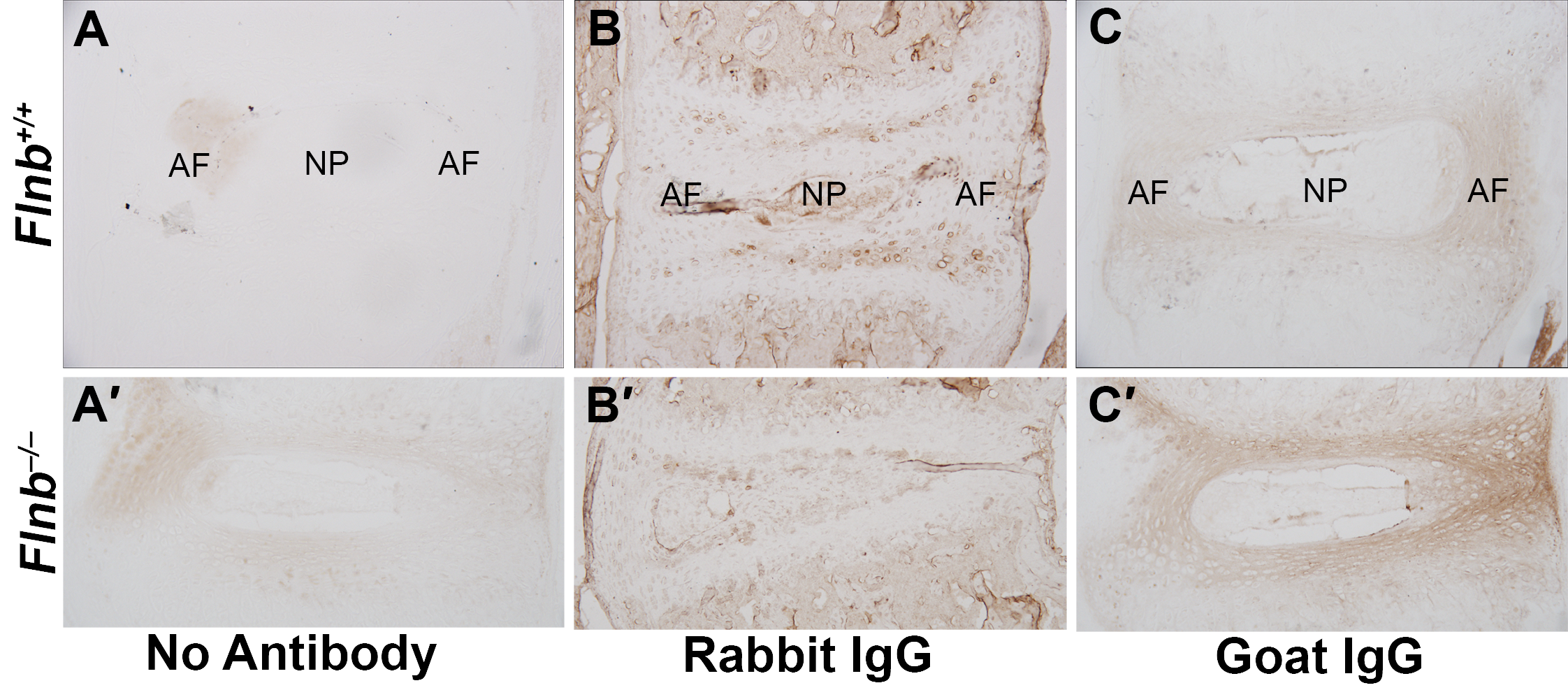

Supplement: S3 Fig — Left: posterior, Right: anterior. (A, A′) Negative control IHC in P15 T7 IVD. (B, B′) IHC using rabbit whole IgG. (C, C′) IHC using goat whole IgG. (TIF) [file pgen.1005936.s003.tif]
